# Soil metabolomics: Deciphering underground metabolic webs in terrestrial ecosystems

**DOI:** 10.1016/j.eehl.2024.03.001

**Published:** 2024-03-20

**Authors:** Yang Song, Shi Yao, Xiaona Li, Tao Wang, Xin Jiang, Nanthi Bolan, Charles R. Warren, Trent R. Northen, Scott X. Chang

**Affiliations:** a State Key Laboratory of Soil and Sustainable Agriculture, Institute of Soil Science, Chinese Academy of Sciences, Nanjing 210008, China; b University of Chinese Academy of Sciences, Beijing 100049, China; c School of Environment and Ecology, Jiangnan University, Wuxi 225127, China; d Institute of Mountain Hazards and Environment, Chinese Academy of Sciences, Chengdu 610299, China; e School of Agriculture and Environment, The University of Western Australia, Nedland, WA-6009, Australia; f The UWA Institute of Agriculture, The University of Western Australia, Nedland, WA-6009, Australia; g Healthy Environments and Lives (HEAL) National Research Network, Australia; h School of Life and Environmental Sciences, University of Sydney, Heydon-Laurence Building A08, NSW 2006, Australia; i Environmental Genomics and System Biology Division, Lawrence Berkeley National Laboratory, 1 Cyclotron Rd, Berkeley, CA 94720, USA; j Joint Genome Institute, Lawrence Berkeley National Laboratory, Berkeley, CA 94720, USA; k Department of Renewable Resources, University of Alberta, Edmonton, Alberta T6G 2E3, Canada

**Keywords:** Dissolved organic matter, Carbon cycling, Metabolomes, Extraction method, Soil microbiome, Rhizosphere ecology

## Abstract

Soil metabolomics is an emerging approach for profiling diverse small molecule metabolites, i.e., metabolomes, in the soil. Soil metabolites, including fatty acids, amino acids, lipids, organic acids, sugars, and volatile organic compounds, often contain essential nutrients such as nitrogen, phosphorus, and sulfur and are directly linked to soil biogeochemical cycles driven by soil microorganisms. This paper presents an overview of methods for analyzing soil metabolites and the state-of-the-art of soil metabolomics in relation to soil nutrient cycling. We describe important applications of metabolomics in studying soil carbon cycling and sequestration, and the response of soil organic pools to changing environmental conditions. This includes using metabolomics to provide new insights into the close relationships between soil microbiome and metabolome, as well as responses of soil metabolome to plant and environmental stresses such as soil contamination. We also highlight the advantage of using soil metabolomics to study the biogeochemical cycles of elements and suggest that future research needs to better understand factors driving soil function and health.

## Highlights

• This review presents the state-of-the-art of soil metabolomics.
• Soil metabolome are closely related with carbon cycling and soil microbiome.
• Soil metabolome are sensitive measures in response to environmental stress.

## Graphical abstract

**Figure:**
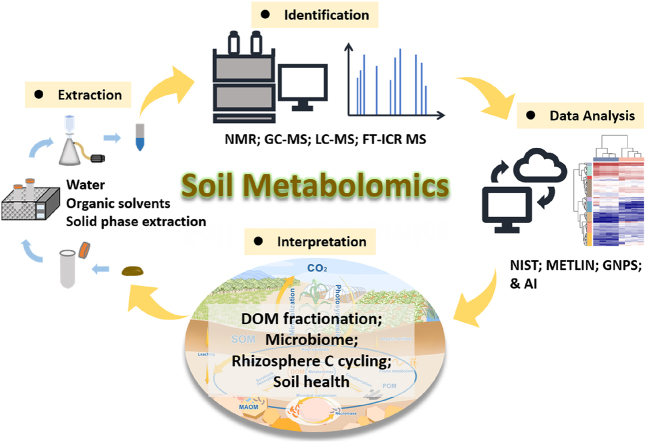

## 1 Introduction

**Fig. 1:**
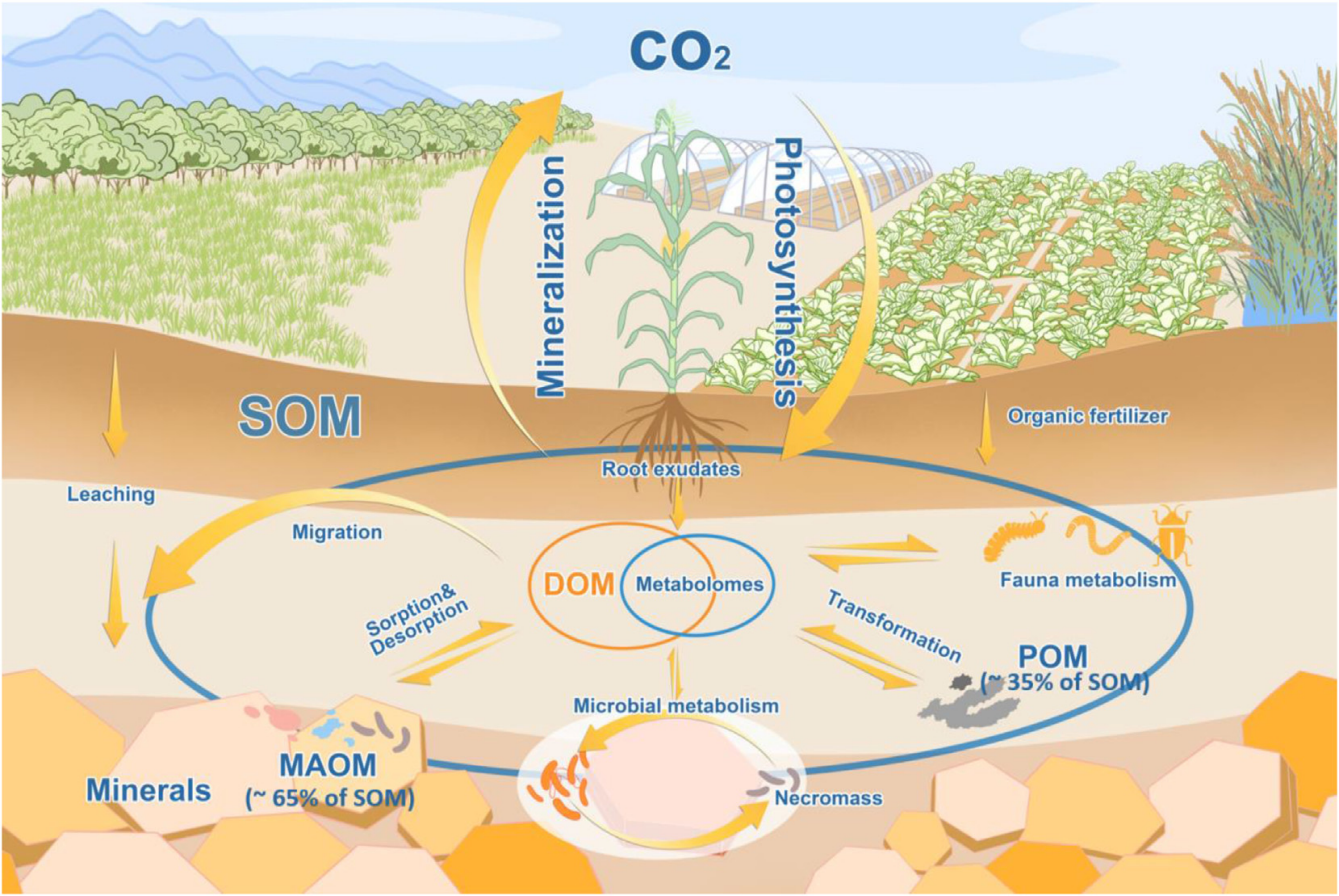
A conceptual diagram showing how soil metabolomics can enhance our understanding of terrestrial carbon cycling. The percentages of MAOM and POM to SOM can be referred to [10]. SOM, soil organic matter; DOM, dissolved organic matter; MAOM, mineral-associated organic matter; POM, particulate organic matter.

The soil is the largest carbon (C) pool in terrestrial ecosystems on Earth [1]. Soil organic matter (SOM), where the soil organic C is stored, is the essential substrate that influences various microbially mediated biogeochemical processes and maintains soil function and health [1,2]. The composition of SOM represents a continuum of progressively decomposing organic compounds and a wide range of organic compounds produced through chemical reactions, microbial synthesis, and root exudation [1,3]. Chemical characterization and fingerprinting of the molecular composition of SOM have always been a research interest for soil and environmental scientists, as SOM makes up the most complex and important substrates in the environment [4]. The SOM is composed of a large number of small molecules derived from plants and microbes, with their abundance and composition being some of the most important mediators of soil health and plant productivity [2,5,6]. Dissolved organic matter (DOM), the water-extractable organic matter fraction in the soil solution that passes through a 0.45 μm filter, constitutes less than 2% of the total SOM [[7], [8], [9]]. However, DOM is the most actively cycling organic matter fraction and influences most of the critical soil biogeochemical cycling processes of major elements, including C, nitrogen (N), phosphorus (P), and sulfur (S), and the conversion and mineralization of SOM [7]. Hence, the metabolism of DOM by soil microbes is a key determinant of soil organic C residence time, and approaches that decrease microbial mineralization could be used for soil C sequestration. In addition, understanding the composition of organic compounds in DOM can improve our knowledge of soil ecosystem services, e.g., C stabilization and transformation, plant growth and food productivity, contamination remediation, and climate change regulation (Fig. 1).

Metabolites are an important component of DOM and can be a direct measure of the biochemical activity of the soil biota. Much biochemical activity happens at the level of individual metabolites, so those compounds are the best measure of biochemical activities [11]. Similarly, the responses of metabolites (at the molecular level) to environmental stress are more sensitive than with traditional indicators such as microbial biomass C (MBC) [12]. Metabolomics, the global profiling of small molecular mass compounds in a sample [[13], [14], [15], [16]], can often detect thousands of metabolite features within a single run (though the number identified is typically a small fraction of this). Metabolomics has found a wide range of applications in the field of environmental science. For example, it has been applied in aquatic ecosystems, including marine and freshwater environments, to elucidate relationships between DOM and microbial communities [17,18]. Soil metabolomics, which focuses on the study of metabolites in the soil, is a relatively new subdiscipline in soil science; however, it is gaining popularity given its ability to provide molecular-level information on C and nutrient cycling and its relationship with soil communities [19,20]. Soil metabolomics has been used in various studies for assessing soil quality and function, including but not limited to studying the biogeochemical cycles of soil C and nutrients, the relationship between metabolites and soil microbes and plants, as well as the responses of soil microbes to environmental stresses and climate change.

Soils contain various compounds produced by plants, microorganisms, and fauna. The small molecule metabolites (<1,000 Da), whether as a direct input or degradation product, are typically rapidly cycled by soil microbial communities, placing metabolites at the heart of C cycling in many ways [[21], [22], [23]]. It is, therefore, important to first understand the range of metabolites found in soils to explore the application of soil metabolomics in soil biogeochemical dynamics. Common classes of soil metabolites include fatty acids, amino acids, lipids, sterols, alcohols, sugars, amino-sugars, sugar alcohols, sugar acids, organic phosphates, aromatics, purines, and other organic acids, etc., as well as many yet-to-be-identified compounds [6,20,[24], [25], [26]]. Ascertaining the source of those compounds presents a big challenge.

The approaches used in soil metabolomics have evolved over the years, with shifts in applications signposted by varying definitions. Early soil metabolomics studies were performed to characterize DOM, e.g., dissolved organic C (DOC) and dissolved organic N (DON), in the soil [20,27,28]. The term “soil meta-metabolome” was proposed by Warren to describe the pool of metabolites directly related to soil processes, such as N and C cycling [27]. Moreover, the metabolic profiles of entire communities, including microbes and invertebrates, living in the soil could be analyzed by “community metabolomics” [29]. Swenson et al. proposed “untargeted soil metabolomics” for the first time and established the working procedure for the measurement of soil metabolites [24]. Then, “exometabolomics” was developed to study the microbial extracellular metabolites in soil science [30,31]. Recently, “environmental metabolic footprinting” has been used to analyze soil metabolomes' response to stresses caused by contaminants [32], and “untargeted rhizosphere metabolomics” has been proposed to analyze entire soil metabolomes [33].

The studies mentioned above constitute the main milestones in the development of soil metabolomics, including the definition, methodology, and interpretation of soil metabolomics. The timelines of these pioneer studies indicate that soil metabolomics developed quickly, from soil metabolite extraction methods and data analysis to the application of metabolomics in various research areas. To the best of our knowledge, there is no comprehensive review focusing on the research progress of soil metabolomics and its applications. Therefore, this paper aims to address the following three aspects: 1) methods for effectively extracting and identifying soil metabolites; 2) new insights into soil biogeochemical cycling gained from soil metabolomics; and 3) emerging research directions in soil metabolomics. At the end of the review, we provide some perspectives on opportunities to greatly improve the performance and impact of soil metabolomics.

## 2 Methodologies in soil metabolomics

Generally, soil metabolomic analysis includes the extraction of metabolites from the soil matrix, metabolite characterization using various analytical instruments, and data analysis and interpretation.

### 2.1 Extraction of soil metabolites

Soils are a highly complex mixture of minerals, biopolymers, biomass, and small molecule substances, often in aggregated structures [34]. Extensive literature exists on the extraction of SOM and DOM/DOC. Soil organic matter extraction methods and the subsequent measurement of extractable SOM can be benchmarked against the total organic C determined through combustion. Common extraction methods include treating soils with potassium sulfate, sodium pyrophosphate, sodium borate, hydroxylamine, hydrochloric acid, and so on, to desorb and dissolve soil organics. Even extreme methods, such as treating the soil with hydrofluoric acid to dissolve minerals, often do not release all of the organic C associated with minerals [[35], [36], [37]]. Critically, all of these salts and acids can interfere with the characterization of soil metabolites when using mass spectrometry. Moreover, some of these procedures (e.g., extraction with sulfuric acid) will change the composition of metabolites, for example, by hydrolyzing biopolymers to form small molecule metabolites [38].

Given these considerations, most metabolomic methods have used chemically “gentle” approaches similar to those used in biomedical and biotechnological applications, especially when using inorganic and/or organic solvents. Aqueous extraction methods have the advantage of extracting the fraction of DOM/DOC that is most microbially accessible. Some of the common extraction methods used in soil metabolomics are summarized in Table 1.

**Table 1:** The extractants and identification instruments used for soil metabolome extraction.

	Extractant	Method of identification	References	Traits
Inorganic solvent extraction	0.01 M and 0.5 M K_2_SO_4_	GC-MS CE-MS	[24,39]	• Suitable for polar metabolites • Simple and reflective of actual microbe-accessible extracellular metabolites • The salt content of the resultant samples complicates metabolite analysis because of salt crystal formation during dry-down and ion suppression • The efficiency of salt-based extractants compared with water is dependent on the ionic strength of the solution and soil properties
	0.01M NH_4_HCO_3_	GC-MS	[24]	
	Water	1H NMR GC-MS CE-MS LC-Q-TOF-MS	[24,28,39,40]	
Mixed organic–inorganic solvent extraction	Chloroform:0.01M K_2_SO_4_ = 1:4 Chloroform:0.5M K_2_SO_4_ = 1.5:100	GC-MS CE-MS	[39,41]	• Suitable for both polar and non-polar metabolites • Extracting both intracellular and extracellular metabolites • The polarity of the extractant determines the polarity of the analyzable metabolites • Organic solvents efficiently extract fatty acids and sterols
	Methanol:chloroform:water = 2.5:1:1, 2:2:1, 2:1:1	1H NMR GC-MS	[29] [42]	
	Isopropanol:methanol:water = 3:3:2	GC-MS	[24]	
	Methanol:chloroform:phosphate buffer = 2:1:0.8	GC-MS	[43]	
	Methanol:0.05% formic acid = 1:1, 19:1	UPLC-Q-TOF-MS	[44]	
	Methanol:chloroform:0.25M NH_4_HCO_3_ = 4:10:5	GC-MS	[25]	
	Acetone:HEPES buffer = 2:1	LC-Q-TOF-MS	[45]	
	Ethyl acetate:water = 1:1	LC-MS GC-MS	[23]	
	Acetonitrile:isopropanol:water = 3:3:2	GC-TOF-MS	[46]	
	Methanol:water = 1:1, 2:1, 3:1, 4:1, 9:1	1H NMR GC-MS HPLC-MS UPLC-Q-TOF-MS	[20,24,33,47,48,49,50,51,52,53]	
Organic solvent extraction	Ethyl acetate	LC-Q-MS	[32]	• Suitable for non-polar metabolites • High frequencies of lipids, as well as amino acids and organic nitrogen compounds, can be detected
	Methanol	GC-MS UPLC-Orbitrap-MS	[54,55]	
	Methanol:ethyl acetate = 3:1	GC-TOF-MS	[56]	
	Methanol:dichloromethane:ethyl acetate:acetonitrile = 1:1:1:1	LC-Q-Orbitrap-MS	[57]	
	Methanol:acetonitrile = 1:1	GC-MS	[58]	
Solid extraction	Tenax: porous polymer resin based on 2,6-diphenylene oxide	GC-MS	[45]	• Suitable for volatile metabolites
	Solid-phase microextraction	GC-MS	[59]	

GC-MS, gas chromatography-mass spectrometry; CE-MS, capillary electrophoresis-mass spectrometry; 1H NMR, H nuclear magnetic resonance spectra; LC-Q-TOF-MS, liquid chromatograph-quadrupole-time-of-flight-mass spectrometry; UPLC-Q-TOF-MS, ultra-performance-liquid chromatograph-quadrupole-time-of-flight-mass spectrometry; LC-MS, liquid chromatograph-mass spectrometry; GC-TOF-MS, gas chromatography-time-of-flight-mass spectrometry; UPLC-Orbitrap-MS, ultra-performance liquid chromatography-orbitrap-mass spectrometry, LC-Q-MS, liquid chromatograph-quadrupole-mass spectrometry; LC-Q-Orbitrap-MS, liquid chromatograph-quadrupole-orbitrap-mass spectrometry.

Soil metabolites include both extracellular and intracellular organic products. Initially, the protocol used for determining total microbial biomass was adopted for extracting metabolites [60]; in this method, intracellular metabolites are extracted by fumigating with ethanol-free chloroform to lyse microbial cells [5]. Accordingly, fumigation of the soil with chloroform significantly increases the abundance of some metabolites. In one study, 2′-deoxycytidine, proline, and thymine were detected only in the fumigated compared to unfumigated soil [24]. By lysing the microbial cells, the fumigation method, in principle, creates a “whole soil extract” that contains both the extracellular and intracellular metabolites [5]. Subsequent studies suggested that chloroform fumigation can create artifacts and result in altered concentrations of some metabolites due to their oxidation via exoenzymes that remain active [61]. Direct extraction with an organic solvent or a water-solvent emulsion avoids artefacts associated with prolonged chloroform fumigation [61,62]. A promising alternative is using solvent-water systems that inhibit microbial activity. For example, low molecular weight organics were several times more abundant in extracts from soil extracted with 0.5 M K_2_SO_4_ that contained 1.5% CHCl_3_ than those extracted with either 0.5 M K_2_SO_4_ or H_2_O [39]. This is presumably due to both improved solubility of target metabolites and cell lysis, resulting in extracts that contain both the extracellular and intracellular metabolites [24]. Common organic solvents include methanol, isopropanol, ethyl acetate, and chloroform, to name a few. In another study, methanol–chloroform–water (first methanol: chloroform = 2:1, and then chloroform: water = 1:1, i.e., the Bligh-Dyer 2-phase extraction procedure) was used to extract polar and nonpolar metabolites, with the advantage of efficiently extracting and separating lipid and water-soluble metabolites [29]. However, it should be noted that some metabolites are likely partitioned in both phases, which can introduce an additional source of experimental error.

As reported in one comparison study, although the abundance (i.e., intensity) of some extracted compounds varied among different extractants, including water, dilute salts (10 mM K_2_SO_4_ or 10 mM NH_4_HCO_3_), as well as organic solvent mixtures such as isopropanol/methanol/water (3:3:2 v/v/v) and 10%, 25%, 50%, 100% methanol in water, the total number of compounds (i.e., diversity) detected in each extraction was similar [24]. Buyer et al. refined the Bligh-Dyer 2-phase method through the inclusion of a volatile salt, i.e., ammonium bicarbonate, to improve the desorption of metabolites from soil minerals and to avoid the interference of the phosphate or citrate used in the traditional Bligh-Dyer 2-phase extraction [25]. As discussed above, salts can be included as part of the extractant to help desorb organics from mineral surfaces; however, high salt concentrations can retain water, interfering with derivatization chemistries used with gas chromatography-mass spectrometry (GC-MS) and resulting in ion suppression during electrospray ionization, depending on their concentrations [19,24].

Generally, water is a good choice for extracting many extracellular metabolites derived from plants or microbes [40], providing information on the composition of the DOC pool with minimal cell lysis, assuming the soil has sufficient salt to avoid cell rupture from osmotic stress. However, organic solvents are preferred for extracting metabolites from microbial biomass and/or analyzing non-polar metabolites such as fatty acids and sterols. The acetonitrile: isopropanol: water solution at a 3:3:2 ratio was used for extracting soil metabolites [6]. A 50% methanol solution extracted more metabolite species from the rhizosphere soil than the water and 95% methanol solution [44]. Moreover, methanol in water (1:1) for extracting polar metabolites, and a 2:1 acetone: 0.2 M HEPES pH 7.7 buffer for extracting nonpolar metabolites have also been proven effective for extracting metabolites from the soil [45]. The diverse extractants used in early studies (Table 1) make it difficult for early career researchers to determine which extractant to use when studying soil metabolomics. More researchers used the methanol/water mixture than other extractants (Table 1). More recently, a protocol was proposed to extract polar and nonpolar soil metabolites with water and a mixture of ethyl acetate: water (1:1), respectively [23]. This will provide guidance to those who are not familiar with the methodologies in soil metabolomics. A standard soil metabolite extraction protocol is the basis for comparing metabolomes from different soils and will be helpful for future global soil metabolite data analysis.

There is growing interest in profiling volatile metabolites in soils, and “the soil volatilome”, which includes compounds such as alcohols, ketones, organic acids, and so on, has been proposed [59,63]. Volatiles are typically captured by passing soil gas through a porous polymer resin based on 2,6-diphenylene oxide (Tenax TA) [45], or extracted by solid-phase micro extraction [59].

In addition to the different extractants used to extract metabolites, there is a diversity of extraction procedures used in soil metabolomics studies, including the single or combined use of sonication [20], shaking [39], vortexing [64], grinding [65], and crushing [66]. Generally, grinding in a partially stabilized zirconia ring and puck bowl, combined with extraction with sonication, breaks up the aggregates and cells, leading to the extraction of both extracellular and intracellular soil metabolites [20]. Compared with sonication and shaking following chloroform fumigation, shaking alone was recommended for untargeted DOM analysis with the smallest loss of soil enzymatic activity indicators [64]. Other less commonly used methods include centrifugation without extractants [27] and microdialysis [67].

Given the diversity of soil types, it is unlikely that there will ever be a single extraction method that has high performance for all soils. Broadly acceptable and cost-effective soil metabolome extraction methods that are suitable for the complete characterization of various components of soil metabolites need to be developed. For a particular soil, sequential chemical extraction methods may be developed to separate extracellular and intracellular metabolites or polar and nonpolar metabolites. For different types of soils, different extraction methods could be developed, similar to the different P extraction methods designed for acidic vs. alkaline soils.

### 2.2 Identification of soil metabolites

The most widely used techniques for profiling soil metabolites are mass spectrometry, such as GC-MS, liquid chromatography-mass spectrometry (LC-MS), and nuclear magnetic resonance (NMR) (Table 1) [68]. The LC-MS and GC-MS techniques have an advantage over NMR methods in characterizing complex and relatively dilute mixtures in profiling soil metabolites [19,69]. However, NMR techniques can determine structures *de novo* and detect metabolites that do not ionize well by LC-MS or GC-MS [70].

The GC-MS technique is well suited for analyzing a wide range of metabolites, including volatiles, typically using electron ionization [59]. Generally, GC-MS has outstanding chromatographic resolution and produces extensive fragmentation information, which is easily compared with reference databases for metabolite identification [5]. However, many metabolites require derivatization prior to GC-MS analysis [43], the yield of which can be affected by water retained in salts [19]. The profile of soil metabolites can be highly correlated with the derivatization technique used, with derivatizations containing methoxyamination solution showing less variability [43]. Nevertheless, many molecules cannot be derivatized (e.g., due to steric hindrance), or are degraded during passage through the hot injection port and column, or have too low a vapor pressure [71]. GC-MS has excellent performance for metabolites that do not require derivatization or are readily derivatized, but the selectivity afforded by derivatization renders GC-MS unable to detect many other molecules.

The LC-MS technique is typically performed either using normal or reversed-phase chromatography [72]. ElectroSpray Ionization (ESI) is used to generate gas phase ions for manipulation using electromagnetic fields, often achieving resolutions > 100,000 full-width half max and mass accuracy < 5 parts per million. Tandem mass spectrometry (MS/MS) is now commonly used to provide additional orthogonal information (in addition to accurate mass and retention time) on the chemical structures of the metabolites [73]. These fragmentation spectra are compared to standard databases such as the National Institute of Standards and Technology, Metabolite Link , and Global Natural Products Social Molecular Networking platform, to provide identifications including spectral relationships to known metabolites or other metabolites in the experiment [74].

Several other techniques are used for soil metabolite analysis. Capillary electrophoresis–mass spectrometry is another technique that has been used to study soil metabolites [27]. It affords exquisite separations of polar, charged metabolites but is not widely used [13]. Recently, proton transfer reaction time of flight mass spectrometry (PTR-TOF-MS) was used to study soil microbial volatile metabolites in response to snowmelt [75], showing a potential non-destructive approach to study the soil biogeochemical cycles. Finally, NMR is becoming increasingly sensitive and able to characterize more complex mixtures [70]. It has, for example, been used to study the response of soil metabolomes to contamination and amendments, such as biochar [20,28,29].

Several studies used multiple soil metabolomic methods and compared their advantages and weaknesses. Van Dam and Bouwmeester found that LC-MS or NMR could be used to analyze water-soluble secondary metabolites [16]. Swenson and Northen [23] and Tang et al. [76] found that LC-MS detected more compounds than GC-MS. However, Jenkins et al. noticed that 11 soil metabolites that were identified by GC-MS were not detected by LC-MS [5]. Sugars, such as hexoses, dihexoses and the sugar alcohol mannitol, would be best identified chromatographically by GC-MS [5]. The LC-MS and NMR were integrated for soil metabolome analysis to analyze sugars and organic acids precisely [77].

The “targeted” data analysis and absolute metabolite quantification of soil metabolites are challenging because they both require the use of authentic standards, which are often not available for many soil metabolites. In “targeted” data analysis, knowledge of the mass spectral properties of standards is used to identify those compounds in soil metabolomics data. Similarly, standards are used to construct calibration curves for absolute quantification. Absolute quantification of metabolite concentrations is challenging because a typical metabolomics experiment might resolve 100 s of metabolites, with concentrations spanning >3 orders of magnitude. Purchasing and analyzing purified standards for 100 s of metabolites is often prohibitively expensive, highlighting the challenge posed by the limited availability of purified standards for many metabolite compounds. It is reported that some of the metabolites with high abundance mass ion signals, including amino acids, sugars, nucleobases, and nucleosides, could be quantified by LC triple quadrupole MS. However, only 25 individual metabolites could be quantified among 96 metabolites [5]. Owing to these challenges, e.g., lack of authentic standards, it is common to use the “untargeted” metabolomics methods for relative quantitation or semi-quantitative analysis using chemically related standards. Thus, limited quantitative work has been performed to identify the fraction of the DOC pool that can be identified using metabolomics. It should be noted that combining untargeted and targeted soil metabolomics would be a promising strategy in soil science. The potentially different metabolites between treatments could be screened by untargeted metabolomics, and the changes of certain metabolites could be verified by targeted metabolomics if standards are available. For example, both untargeted and targeted soil metabolomics were combined to successfully elucidate the eco-corona formation of soil metabolites on the surface of microplastics [78].

## 3 Research progress in soil metabolomics

### 3.1 Metabolomics for characterizing SOM and for understanding the behavior of DOM

There is a notable overlap between DOM and metabolome in soil (Fig. 1). Soil metabolomics focuses on the measurement of the composition of compounds with low molecular weight (e.g., sugars, fatty acids, and amino acids) in DOM, which helps elucidate the roles of metabolites in SOM stabilization and mineralization, and the transformation of SOM-associated nutrients. Therefore, the early stage application of soil metabolomics focuses on the characterization of the molecular composition of DOM in soil [24,27,28,39,79,58]. For example, it was discovered that the pool of N-containing small organic compounds was more diverse than generally recognized, including abundant quaternary ammonium compounds in addition to the well-known amino acids [27].

Metabolomics can provide insights into changes in DOM composition even when there is no change in overall DOM concentration (total C or N). For example, adding engineered nanomaterials to the soil did not change total DOM concentration but altered the composition and redistribution of soil metabolites in DOM [47]. The amendment of biochar to the soil indeed decreased carbohydrate and peptide concentrations in DOM, even though the total concentration of DOM increased [28]. Thus, soil metabolomics can provide a higher resolution of the changes of specific compounds in DOM in response to environmental conditions than that conducted with the measurement of overall DOM concentration. Hassanpour et al. also stated that the metabolomics-based analysis could help with high-resolution evaluation of the spatial divergence of the DOM in water [80]. Fourier transform ion cyclotron resonance mass spectrometry (FT-ICR MS), an ultrahigh-resolution electrospray, has been used to characterize DOM [68,81,82]. Unlike metabolomics analysis that typically uses LC-MS/MS to identify metabolite features <1,500 Da—a fraction of which is definitively identified—FT-ICR MS typically does not use chromatography but instead takes advantage of the ultra-high resolution to directly assign chemical formulas for all of DOM with a broad range of molecular sizes [68]. A few limitations of FT-ICR MS are that this technique typically stops at the level of identifying chemical formulas, which is less available, hard to operate, and more costly than LC-MS or GC-MS.

Sorption of DOM by minerals is an important abiotic factor affecting the environmental behavior of DOM in the soil. Soil metabolomic analysis provides the fingerprinting of the redistribution of various metabolites of DOM in soil solution (i.e., pore water) and solid (i.e., adsorbed on soil minerals) phases. Bacterial lysates, such as phosphate-containing and dicarboxylate metabolites, could be strongly sorbed by clay minerals, including ferrihydrite, thereby influencing the mobilization of phosphate [21]. The sorption and desorption of soil metabolites on Fe oxides are quite different from that on silicate minerals such as kaolinite [79]. Using ^13^C-labeled lysates from a soil bacterium, Swenson et al. found that cationic and anionic metabolites had the lowest recoveries, while non-ionized metabolites exhibited high recovery after the lysates interacted with sterilized soil [24]. This indicates that the sorption of metabolites to soil may cause the under estimation of DOM fractions in the soil. As mentioned above, the desorption of metabolites from minerals can be achieved with an extractant of high ionic strength. More generally, in the last 50+ years, soil chemistry has developed means to extract different soil pools preferentially by altering extractant chemistry. These range from complex sequential extraction procedures yielding three or more operationally defined pools (e.g., sequential extraction scheme for soil P [83]) through simple contrasts such as extraction with water versus 2 M KCl to quantify free and free + adsorbed pools, respectively. Soil metabolomics should capitalize on this wealth of knowledge; however, classic soil chemistry approaches are not readily compatible with mass spectrometry because they involve inorganic salts, sometimes in high concentrations (e.g., 0.5 M K_2_SO_4_, or 2 M KCl) and sometimes in complex mixtures (e.g., the popular Mehlich-3 extract, which uses a combination of acetic acid, ammonium nitrate, ammonium fluoride, nitric acid, and ethylenediaminetetraacetic acid). Therefore, future research needs to focus on developing mass spectrometry-compatible extraction techniques to analyze metabolite distribution in both the solution and solid phases of the soil.

### 3.2 Soil microbiome and metabolome

There is a significant relationship between soil metabolome and soil microbiome, the latter being defined as a characteristic microbial community occupying habitats in the soil and referring to microorganisms and their theater of activity [84]. Thus, this relationship includes but is not limited to i) the contribution of microbial metabolites to soil metabolome, ii) the networks and correlation between soil metabolites and microbial community composition, and iii) the relationship between soil metabolites and microbial community function, activity, etc. Soil microbes play a major role in the biogeochemical cycling of elements, including plant nutrients such as C, N, P, and S, and the cycles of soil organic contaminants and heavy metals; in addition, microbial metabolites make up a significant part of soil metabolomes (Fig. 1). Soil metabolites in DOM with molecular weight lower than 1,000 Da are the most accessible growth substrates for soil microbes [5]. It is difficult to assess the total contribution of soil organics that are derived from microbial metabolites. However, a current view is that much of soil organics originate from microbial necromass [2,85]. It has been estimated that soil microbial metabolites can account for more than 15% of the total MBC [41]. Since microbes use small molecules directly as substrates and different groups of microbes target different subsets of metabolites, the abundance, diversity, and availability of soil metabolites will likely affect the structure, activity, and function of soil microbial communities [31]. Researchers are increasingly exploring the relationship between soil microbiomes and metabolomes to better understand soil biogeochemical cycling processes, especially the C cycle in relation to soil C stabilization and sequestration [19], nutrient cycles in relation to bioavailability and nutrient flux in soil [40,44], and contaminant cycles in relation to soil remediation [86].

Metabolites occupy a central position in C stabilization in soil. There continues to be active debate about the mechanisms of soil C stabilization/preservation. One common view is that simple organic compounds that are converted by microbes into microbial necromass (which often includes waste products) contribute to the stable C content in the soil [87]. Necromass and metabolites can be stabilized via physicochemical associations with clay minerals (such as Fe and Al oxides, and silicates), resulting in what is referred to as mineral-associated organic matter (MAOM, accounting for approximately 65% of SOM [8,9,10], Fig. 1). In the case of MAOM, the interaction of microbial metabolism with soil mineralogy determines the proportion of plant inputs and microbially derived C that are incorporated into the MAOM and get stabilized in soil (i.e., C sequestration).

Sequencing technologies have enabled extensive investigation of soil microbial community structure [88,89]. The use of multi-omics, including metagenomics and metabolomics, has also been expanded to study soil microbial community networks [90]. Metabolic network analysis is one of the complementary tools that critically aid in exploring cross-species metabolite exchanges and the activities of non-culturable microbial populations in the soil [91]. The relationships between soil microbes and soil metabolites can be examined using techniques such as Procrustes tests combined with Mantel tests [48], or other network correlation analyses. For example, it was shown that during the wetting of soil biocrust, microbes of *Microcoleus* sp. released metabolites such as adenosine and adenine. At the same time, those in *Bacillus* sp. consumed metabolites such as glutamate and myristate [54]. More recently, the application of machine learning to these data sourced from desert soil biocrust wetting [54] helps identify additional associations between soil microbes and metabolites [92]. Significant positive or negative co-occurrences based on Pearson correlation exist between soil metabolites and certain microbes [93]. Similar approaches have been used to show that sucrose had the greatest correlation with bacterial members in the rhizosphere soil of green pepper [56] and that cerium dioxide (CeO_2_) nanoparticles altered the co-occurrence network between soil microbial communities and metabolites [42]. Metabolic cross-feeding between microbes, i.e., microbes using metabolites of other members to maintain their own growth in the environment [94], is an area of intense interest and is suggested by positive or negative correlations between soil metabolites and certain microbes [45,49]. It is important to note that there may be uncertainties in the correlation between the soil metabolome and microbiomes. These uncertainties could arise from the lack of pure strain or standard metabolite. Thus, stable isotope-based technologies are needed to test these predicted relationships under field conditions.

Understanding the relationships between soil metabolites and microbial activity and function can help design interventions [28]. For example, anaerobic soil disinfestation, which creates anaerobic soil conditions through the incorporation of easily decomposable soil amendments, application of plastic mulch, or irrigation to form saturated soil conditions, reduces soil metabolites, resulting in changes in microbial metabolic pathways, including pyruvate metabolism, glycolysis, butanoate metabolism encompassing tricarboxylic acid cycle [40]. Environmentally relevant concentrations of testosterone can downregulate the microbes' amino acid metabolism processes and decrease the contents of soil metabolites such as isozeaxanthin, hydroxyatrazine, and l-isoleucine [48]. The carbohydrate metabolism of soil microbial communities was found to be promoted by sulfadiazine antibiotics, which may reduce the content of carbohydrates, thus reducing the protection for SOM [95]. Recently, Yang et al. observed that plant growth-promoting microbes such as *Arthrobacter ureafaciens* DnL1-1 and *Trichoderma harzianum* LTR-2 induced a specific wheat rhizosphere metabolite pool, including upregulated lipids, benzoic acids, and amino acids [50], suggesting that microbial metabolism can be a sensitive indicator of environmental changes [96,97]. Lu et al. recently found that metabolomes reach equilibrium faster than soil microbial community structures in response to the cultivation of woody species [96].

Tracing the fate of metabolites during microbial metabolism in the soil is difficult because of their transient flux, complex cycling with multiple sources and sinks for many metabolites, and various compounds in the soil that may interfere with the analysis. The fate of some metabolites can be deduced via isotope label approaches. For example, where labeled isotopologues are available, one can determine bidirectional fluxes of metabolites across plant roots [98], while the flux of metabolites through soil solution can be determined via an isotope pool dilution experiment [99]. Inputs of metabolites to the soil via plant root exudates can be deduced from pulse labeling of ^13^CO_2_ incorporated during photosynthesis appearing in the rhizosphere [100]; however, most studies have examined total rhizosphere ^13^C rather than incorporation into individual metabolites [101,102]. Soil exometabolomics, also known as “metabolic footprinting” [103], is a powerful approach for studying the metabolism of a set of small molecule compounds by the microbial community or microbial strains under laboratory conditions [30]. This method uses metabolomics to compare inoculated vs. uninoculated media to directly explore the causal relationship between microbiomes and metabolites under relatively simple conditions that avoid external environmental interference, e.g., a metabolite is produced or consumed by a microbe. It was observed that soil bacteria have different substrate preferences, suggesting that exometabolite pools contribute to microbial diversity and community structure [31]. Some efforts have been made to create soil-relevant defined media that enable complete tracking of the various organic metabolites, including a soil-defined medium [5] and, more recently, a new medium that takes elemental stoichiometry into account and has been shown to support the growth of diverse soil microbes [104]. Using soil exometabolomic footprinting methods, Cyle et al. found that there was no clear relationship between the compounds' nominal oxidation state of C and the order of substrate depletion by a pure strain, *Paraburkholderia* sp. 1N, which belongs to copiotroph [77]. They further confirmed the complexities of the metabolomic strategies of microbial communities in metabolite cycling in soils [105]. The accumulation of the short-chain carboxylic acid in the soil anoxic incubation condition was found to be greater than that in the oxic condition by measuring the metabolism of ^13^C-glucose through the exometabolomics approach [106]. We anticipate that large-scale exometabolite profiling of soil microbes in combination with metagenomic sequencing will significantly help predict metabolic niches and interactions [107].

### 3.3 Rhizosphere soil metabolomics

The rhizosphere is the most dynamic interface between the soil and the plant, with a large amount of substrates and much energy flowing through the soil, forming a hotspot for soil nutrient transformation and biological activity [108]. About 10%–40% of plant photosynthetic products are released into the rhizosphere soil as root exudates [109]. However, the diversity of compounds released in the soil rhizosphere remains poorly understood. The rhizosphere chemicals contain root exudates and products from their breakdown, microbial metabolites, and compounds derived from biogeochemical cycles of various products such as those released through the decomposition of SOM (Fig. 1). Hence, studying metabolites of the rhizosphere can be challenging because many metabolites can have multiple sources and sinks. These compounds can be studied by untargeted rhizosphere metabolomics [33], while isotope labeling techniques can be employed to study fluxes [98,99]. Rhizosphere soil metabolomics has been used to study the distribution and speciation of root exudates, root–microbial interaction, and the response of roots to both abiotic and biotic stresses, including exposure to toxic contaminants [16]. Zhalnina et al. used exometabolomic analysis of root exudates and demonstrated that bacteria enriched in the rhizosphere preferred to use aromatic organic acids [109].

Metabolomics analysis of plant root exudates helps better understand metabolites' role as signals between plant roots and nematodes, microbes, and competing plants. Metabolomics analysis showed that flavonoids, lipids, and alkaloids were enriched in *Arabidopsis* soil, and compounds that were not annotated were more abundant in non-rhizosphere than in rhizosphere soil [44]. By analyzing the variation of metabolites from different genotypes of sorghum (*Sorghum bicolor*) growing in soil, clay, or sand, it was shown that belowground substrates influenced profiles of root exudates and that sucrose was greatly enhanced in the rhizosphere environment [110]. Seven soil metabolites were significantly higher in the soil with alpine meadow plants growing on *Floccularia luteovirens* fairy rings compared to those outside of the fairy rings [111]. Moreover, the rhizosphere metabolites with maize (*Zea mays*) grown in soil differed from those grown in water incubation experiments [33].

Rhizosphere exudates are closely and dynamically connected with soil microbes that reside within the rhizosphere [109]. For example, a negative correlation has been observed between the carbohydrates and the soil bacteria of *Chloroflexia* and *Actinobacteria* in the rhizosphere of *Camellia oleifera* [96]. It has also been found that iron-oxidizing bacteria could change the distribution of soil metabolites in rhizosphere soil [66], while some of the key metabolites were consistent with those in non-rhizosphere soil. Song et al. measured metabolites from the pepper rhizosphere and bulk soils under plastic greenhouse vegetable cultivation and found that starch and sucrose metabolism pathways varied the most between rhizosphere and bulk soils, with downregulation of the functional genes participating in this pathway in the rhizosphere [56]. Concentrations of trehalose and betaine, as well as choline-like and carnitine-like compounds detected by NMR, and amino acids such as aspartic acid and glutamine detected by LC-MS, were higher in rhizosphere soils of *Burkea* tree than those in non-rhizosphere soils [112].

A major challenge in interpreting rhizospheric metabolomic data lies in differentiating between metabolites produced by the plant host vs. rhizosphere bacteria [69]. The limited availability of data on the quantity of metabolites further complicates the understanding of the dynamic of the rhizosphere metabolites. It has been suggested that metabolomics may not be able to resolve all signaling relations in rhizosphere soil [16]. Thus, using a metabolomic approach, the term “signalomics” has been proposed to describe the chemical communications between plants and microbes and among microbes themselves in the rhizosphere [108].

### 3.4 Soil metabolomes and soil contamination

The response of soil metabolome to contamination has been identified as a key ecological indicator and molecular marker in soil remediation. Soil contamination by persistent organic pollutants, potentially toxic elements, and emerging contaminants such as nanomaterials and microplastics has become a serious environmental issue. The response of individual biota or entire communities to contaminants could be predicted or assessed by sensitive compounds, i.e., biomarkers [113]. Recently, soil metabolites have been investigated as biomarkers for soil contamination. The least variation in the soil's entire community metabolites occurs in soils with similar pollution profiles based on principal component analysis [29]. Patil et al. proposed environmental metabolic footprinting (EMF) to analyze soil metabolomes in response to the stress of contaminants, such as herbicides [32]. They found that the impact period of leptospermone, the natural β-triketone herbicide, on the soil microbiomes was shorter than that of systemic post-emergence herbicides, e.g., sulcotrione [32]. EMF can be used to generate both endogenous and exogenous metabolite profiles that can serve as biomarkers for contaminants in soils. For example, EMF reflected more information, e.g., the by-product formation for pesticides and microbial metabolism modification, than the measurement of the half-life of pesticides in the environment [114]. In a case study, phytosphingosine was detected as a specific metabolite in soils contaminated with phenol and hydrogen fluoride [55]. The responses of soil metabolomes to the contamination of polycyclic aromatic hydrocarbons (PAHs) [93], chlorpyrifos [115], epoxiconazole [116], and CeO_2_ nanoparticles in soil [117] were also investigated to elucidate the soil microbial response to the stress of contaminants. Starch and sucrose metabolism, N metabolism, S metabolism, propanoate metabolism, fatty acid metabolism, and the urea cycle were fluctuated in soil exposed to CeO_2_ nanoparticles, resulting in enhanced concentrations of fatty acids, amino acids, and amines [42].

Additionally, the rhizosphere acts as a hotspot for the remediation of soil contaminants. The C metabolism has been found to affect the degradation of PAHs in the rhizosphere [86,118]. The rhizosphere metabolites, including levoglucosan, linolenic acid, 4-hydroxycinnamic acid, and allo-inositol, were significantly increased in response to the addition of engineered nanomaterials such as silicon dioxide (SiO_2_), titanium dioxide (TiO_2_), or ferroferric oxide (Fe_3_O_4_), in soils planted with maize [47]. Similarly, metabolites (including sugars and sugar alcohols, fatty acids, and small organic acids) increased, while amino acids decreased in the *Brassica rapa* subsp. *chinensis* (Pakchoi) rhizosphere soil exposed to SiO_2_ nanoparticles [51]. The effect of Ag nanoparticles on the soil metabolite profiles in soil planted with cucumber was also studied, showing that the negative impact of Ag nanoparticles on microbes, such as decreasing the abundances of fatty acids, was not altered by the presence of the plant [52].

Microplastic contamination in terrestrial environments is an emerging concern. Polybutylene succinate and polylactide microplastics were reported to alter the lipid metabolism in soil [119]. Polyethylene microplastics significantly changed the soil metabolite spectrum in a short period, including organic N compounds, lipids and lipid-like molecules, and organic acids and derivatives [120]. The coexistence of oxytetracycline and polyethylene microplastic in soil significantly increased the relative concentrations of arachidic acid and indoleacetic acid while decreasing dodecanoic and heptadecanoic acids [121]. It remains to be clarified whether there are dose–effect relationships between certain soil metabolites and contaminants. Further research is essential to test whether soil metabolomes could act as molecular-level biomarkers sensitive to soil contamination. The impact of the contaminants of emerging concern on soil metabolomes needs to be further studied for potential risk assessment in the future.

### 3.5 Metabolomics for the analysis of soil health and climatic stresses

Soil metabolomes can be a sensitive indicator of soil quality. Soil metabolome has the potential to provide more sensitive soil quality assessment than the traditional soil quality indicators, including pH, cation exchange capacity, SOM, MBC, enzyme activity (e.g., dehydrogenase), and so on [6]. For example, it was reported that the soil metabolites tend to cluster better to land use than the location of sampling, contrasting with findings based on soil chemical elemental composition [20]. Similarly, Nguyen et al. measured soil metabolomes from 188 backyard soils across 14 U.S. States, demonstrating that soil metabolomes could reflect the effects of local factors such as temperature, light level, and human activities on the soil [57].

The responses of soil microbes to climatic stress and soil management can also be characterized by soil metabolomics. The soil metabolism process was strongly impacted by climate change-induced extreme weather events, including drought [46]. Kakumanu et al. found that the soil metabolites such as sugars and alcohols did not increase consistently with soil drying [41]. The drought-stressed soil contained >10-fold more known microbial osmolytes than those in the control [39]. Soil microbes could enhance the production of osmolytes, such as accumulating sugars and polyols, to alleviate environmental stresses [122]. The impacts of global warming [53], drying and rewetting [99], and P fertilization [123] on the soil metabolomes have also been studied. Therefore, characterizing the changes in soil microbiomes will further elucidate the responses of soil microorganisms to climate changes in the future.

## 4 Conclusions and future perspectives

**Fig. 2:**
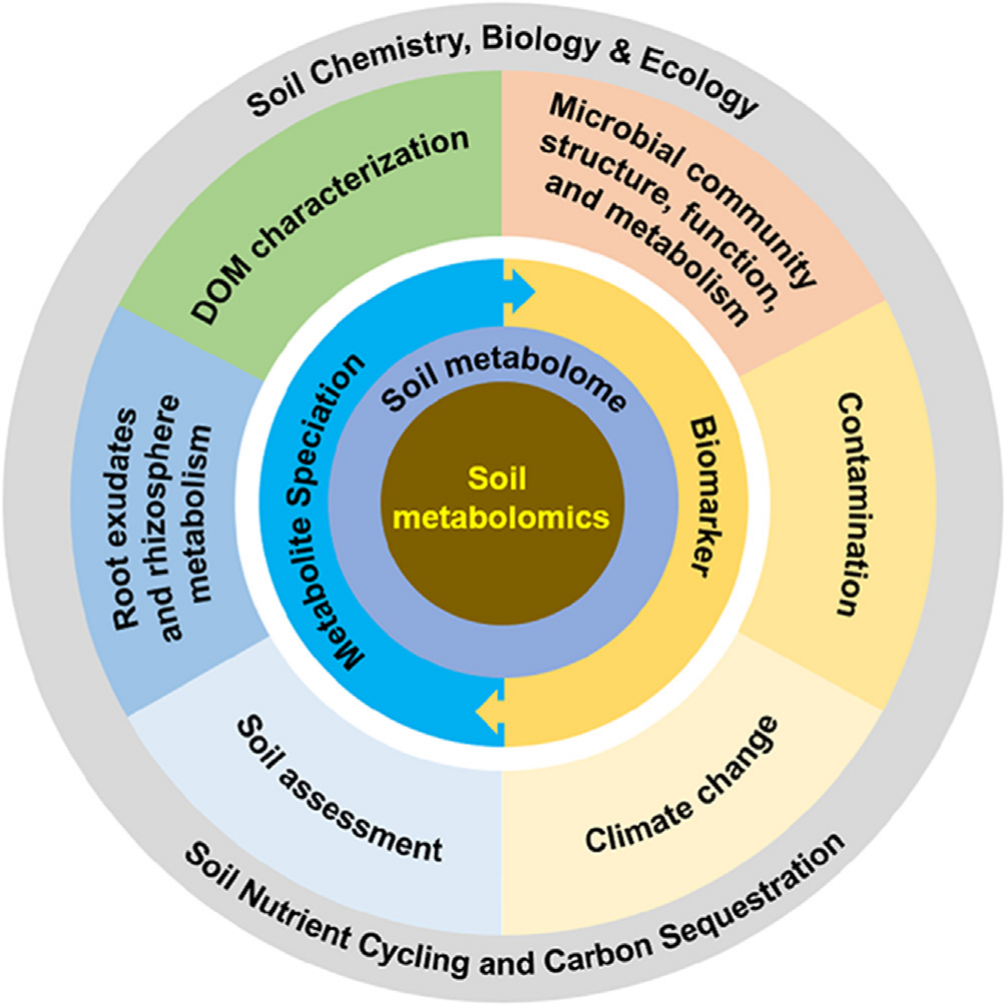
The research progress and application of soil metabolomics. Soil metabolomics studies the abundance, diversity, composition, and dynamics of soil metabolome in response to temporal and spatial variations of soil conditions. Soil metabolites can act as biomarkers for stresses such as climate change and environmental contamination. In the future, the relationship between soil metabolome and microbial community and the inherence of soil metabolome and nutrient cycling and carbon sequestration will be broadly considered, and soil health assessment based on soil metabolome will be developed.

Soil metabolomics has developed rapidly, attracting growing interest for its application in researching soil biogeochemical cycles. Studies on the characterization of DOM, microbe–plant interaction in the rhizosphere, and microbial response to environmental stress and climate changes have been conducted via soil metabolomics, using nontargeted or targeted techniques. Soil metabolomics is a powerful approach connecting soil chemistry, soil biology, and soil ecology (Fig. 2), poised for further development along with new techniques and instruments. In the following section, we highlight a number of areas of standardization and research that we believe will greatly improve the performance of soil metabolomics.

1) Standardization of methods and reporting

The metabolomics field, including its subfields such as soil metabolomics, has a well-acknowledged need for standardization [[124], [125], [126], [127]]. For example, an urgent need for standardization can be observed in the area of soil extraction methods, which are highly variable between labs. A plethora of methods have been used in different labs to extract metabolites from the soil (Table 1). Since the metabolites detected depend on the exact extraction method, standardization is needed to enable cross-study comparisons, for example, to enable global data comparison in soil science and the development of consistent bioindicators [19]. With this said, we also acknowledge that there is a need for improved extraction methods, especially for soils with high clay contents as well as soil components such as MAOM. Given the diversity of global soil types, we envision the development of standard extracting methods tailored to different soil types with different SOM, clay mineralogy, pH, etc.

2) New metabolites need to be identified

Most compounds detected in metabolomics experiments are not assigned (estimated to be <14% [128]), particularly in less studied systems such as soils. This greatly hampers the understanding of the entire soil metabolome and their roles and relationships with soil microbes, plants, and biogeochemical cycling of elements. We emphasize the need to expand databases and cheminformatic tools for assigning the chemical structure of metabolites, a step critical to improving the performance of soil metabolomics. We further anticipate that the application of artificial intelligence will facilitate the analysis of mass spectrometric data for the identification of metabolites. For example, a recently report method employed machine learning to determine chemical formulas based on fragmentation data, enabling conventional instruments to provide information comparable to those obtained using more specialized FT-ICR instruments [129].

3) Gaining new insights into mechanisms of soil C stabilization

We anticipate that soil metabolomics will become increasingly important, given the significant interest in using soils to sequester carbon. This is because plants and microbes produce and act on specific chemical structures (e.g., glucose vs. galactose), and therefore, in many cases, it will be important to characterize SOM and DOM at the molecular level. It will be important to develop targeted methods (likely using isotopic standards) to determine the absolute quantities of metabolites in the soil, for example, by using stable isotope additions [130]. In the future, the high-resolution analysis of the DOM, coupled with targeted soil metabolomics, will greatly deepen our understanding of the components and dynamics of DOM in the soil. Moreover, since, in general, MAOM contributes the largest fraction of SOM, further research on the molecular composition of MAOM should be conducted with soil metabolomics to understand the contribution of small molecular compounds to the formation of MAOM. As described above, this will require new methodologies for soil MAOM extraction that are compatible with the next generation of molecular identification techniques.

4) Isotope-based metabolomics

Using isotope-labeled metabolites to study their transformations will help expand research capabilities, especially for those metabolites that are released in trace amounts and are rapidly degraded in the soil [131]. For example, ^13^C [100], ^15^N [132], and D_2_O labeling [133] have been used to study the fluxes of root exudates, gross fluxes of substrates, and *de novo* synthesis of metabolites in soil. Isotope-based metabolomics will be a powerful technique to elucidate soil metabolites' environmental fate and behavior in future research [106]. These can also provide important insights into the flux of metabolites within soils [99,130].

5) Investigation of spatiotemporal dynamics of soil metabolome

The soil metabolite profile is very dynamic. However, most reported studies are based on soil metabolite profiles at selected sampling points. The spatial and temporal changes in the concentration and composition of metabolomes should be linked with the soil's biochemical properties and environmental conditions. The relationship between soil metabolomes and microbiomes, especially in response to climate change, needs further research. The dynamics of soil metabolomes will provide new insights into interactions among soil biota, such as microbe–microbe, microbe–environment, and microbe–plant interactions through chemical dialogue or food web [94].

6) Exometabolomic profiling of soil microbes using big-data

With the development of molecular biology and machine learning techniques, exometabolomics may be extended from culturable to unculturable microbial strains or communities, allowing us to identify their substrate preferences and secreted products to help interpret the metabolic web of microbes in soils. The integration of exometabolomics and spatial information through techniques such as mass spectrometry imaging will be another powerful method for elucidating microbial interactions in the soil.

7) Multitechnology approaches in soil metabolomics

Both NMR and MS data could be combined and used to analyze soil metabolites. Combining multiple analytical methods for metabolomics, such as NMR and GC-MS, has been suggested [70]. Employing multi-omics tools to study the transformation and stabilization of SOM is an emerging aspect. Metabolomics could verify genomics results to indicate what has happened in the soil driven by microbes. Therefore, integrating soil metabolomics with metagenomics, metatranscriptomics, and metaproteomics, coupled with machine learning technology, should be used broadly to deepen our understanding of soil biogeochemistry and their responses to management and climate change in the future.

## CRediT authorship contribution statement

Y.S. and S.X.C. developed the idea for this manuscript. Y.S. organized the manuscript. X.J. and S.X.C. supervised this work. Y.S., S.Y. and X.L. designed the figures. T.W., N.B., C.W., T.N edited the manuscript and made substantial contributions. All authors discussed and reviewed the manuscript.

## Declaration of competing interests

The authors declare no competing financial interest.
